# A C_2_-Symmetric Biphenyl-Bridged Bis-Aurone: Synthesis, *Z*/*E* Photoisomerization, and Conformational Insights from NMR Spectroscopy and DFT Analysis

**DOI:** 10.3390/molecules31213963

**Published:** 2026-11-03

**Authors:** Maria Antonietta Dettori, Davide Fabbri, Roberto Dallocchio, Nicola Culeddu, Emanuela Azara, Paola Carta

**Affiliations:** Istituto di Chimica Biomolecolare, Consiglio Nazionale delle Ricerche, Traversa La Crucca 3, I–07100 Sassari, Italyemanuelagigliola.azara@cnr.it (E.A.); paola.carta@cnr.it (P.C.)

**Keywords:** biphenyl-bridged aurones, *Z*/*E* photoisomerization, NMR spectroscopy, conformational dynamics, DFT calculations, magnetic anisotropy

## Abstract

Aurones are naturally occurring flavonoids characterized by a benzofuran-3(2H)-one scaffold bearing an exocyclic benzylidene double bond. Herein, we report the synthesis and NMR-based structural characterization of a highly oxygenated biphenyl-bridged bis-aurone and its mono-condensation derivative. UV irradiation at 254 nm in deuterated acetone generated the corresponding E-containing photoisomers, whose formation was monitored by ^1^H NMR spectroscopy. For the bis-aurone, weak additional resonances indicated a minor species possibly corresponding to the doubly isomerized (E,E′) form. NOESY correlations provide constraints on the spatial arrangement of the bis-aurone in solution. Both isolated E-containing derivatives exhibit a pronounced downfield shift of one biphenyl aromatic proton. Conformational analysis is used to examine possible geometrical and magnetic-anisotropy contributions to this observation. The study focuses on synthesis, NMR observations of *Z*/*E* photoisomerization, and conformational interpretation; intrinsic photochemical efficiencies were not determined.

## 1. Introduction

Aurones constitute a distinctive subclass of flavonoids, readily identified by their benzofuranone framework conjugated with an exocyclic arylmethylene unit. Together with chalcones, aurones belong to the anthochlor family of yellow flower pigments, although they occur much less frequently in nature [1,2,3,4]. While chalcones are widely distributed throughout higher plants, aurones are responsible for the characteristic bright golden-yellow coloration of several ornamental flowers and make an important contribution to floral pigmentation. Interestingly, the names of both classes reflect their characteristic colours: *chalcone* derives from the Greek *chalkós* (χαλκός), meaning “copper” or “bronze”, in reference to the yellow orange to bronze hues of many naturally occurring chalcones, whereas *aurone* derives from the Latin *aurum*, meaning “gold”, referring to the intense golden-yellow colour typical of these pigments. Their distinctive molecular architecture, combining an extended π-conjugated system with a rigid benzofuranone scaffold, has attracted considerable attention owing to a broad range of biological activities, including antioxidant, antimicrobial, anti-inflammatory and anticancer effects [2,5,6]. Besides their biological relevance, aurones have recently emerged as attractive photoresponsive molecules. Their conjugated benzofuranone–benzylidene framework can undergo reversible *Z*/*E* photoisomerization, and appropriately substituted derivatives have been investigated as visible-light photoswitches, fluorescent systems and potential platforms for photopharmacology [7,8,9].

Structural modifications that extend conjugation or alter molecular rigidity may substantially influence both photochemical behaviour and spectroscopic properties. Among the strategies used to modulate the properties of bioactive and photoactive molecules, covalent dimerization represents an attractive approach, as it can increase molecular size and rigidity, extend the conjugated framework, and modify the electronic and functional interplay between the two molecular units [10]. Biphenyl scaffolds are particularly interesting in this context because the two aromatic rings are electronically coupled while retaining conformational flexibility through rotation about the central biaryl bond.

In a previous study [11], our group reported the synthesis and antioxidant evaluation of a series of oxygenated phenolic compounds, among which compound (*Z*,*Z*’)-**1b** was the only aurone-based derivative (Figure 1). Although structurally related to the present compound, this compound is a different dimeric aurone architecture in which the linkage between the two aurone moieties differs from that investigated here. Related aurone-containing dimers and alternative linkage patterns provide structural context; the present compound features two aurone units attached to a common biphenyl framework.

Herein, we report the synthesis and structural characterization of (2*Z*,2′*Z*)-2,2′-((5,5′,6,6′-tetramethoxy-[1,1′-biphenyl]-3,3′-diyl)bis(methanylylidene))bis(5,6-dimethoxybenzofuran-3(2H)-one), (*Z*,*Z*′)-**1a** (Figure 1), together with its mono-condensation derivative (*Z*)-**1c** (Scheme 1). To the best of our knowledge, the title compound represents the first C_2_-symmetric bis-aurone in which two aurone chromophores are directly connected through a biphenyl framework.

The photochemical behaviour of both compounds was investigated under UV irradiation by ^1^H NMR spectroscopy to assess how incorporation of two photoresponsive aurone units within a single biphenyl scaffold affects *Z*/*E* isomerization. Particular attention was devoted to the structural and conformational consequences of photoisomerization. Here, the term “C_2_-symmetric” refers to the constitutional equivalence of the two halves of the (Z,Z′) molecular framework and does not imply exact C_2_ point-group symmetry for any individual solution conformer. The photogenerated (*E*,*Z*)-**1a** derivative displayed an unusually downfield-shifted biphenyl proton at approximately δ 8.7–8.9 ppm, a spectroscopic feature also observed in the corresponding (*E*)-**1c** derivative and previously reported for several *E*-aurones [8].

By combining multidimensional NMR spectroscopy with DFT conformational analysis, we investigated the origin of this anomalous resonance and the conformational flexibility associated with rotation about the central biaryl bond. The analysis examines a possible structural link between aurone *Z*/*E* photoisomerization, three-dimensional molecular reorganization, and magnetic anisotropy in this biphenyl-bridged bis-aurone framework.

## 2. Results and Discussion

### 2.1. Synthesis of Title Compound

The target bis-aurone derivative (*Z*,*Z*’)-**1a** was synthesized through a double Knoevenagel condensation between the methylated dimer of bisvanillin **2** and 5,6-dimethoxybenzofuran-3(2H)-one **3** using neutral alumina as heterogeneous catalyst in dichloromethane solution (Scheme 1). This protocol, previously reported for the preparation of aurone derivatives, proved to be an efficient approach for constructing the highly oxygenated C_2_-symmetric molecular framework. The bifunctional nature of precursor **2**, containing two aldehyde groups, enabled the sequential formation of two exocyclic benzylidene double bonds, affording the bis-aurone derivative as the main product. Alongside the desired bis-condensation product, the mono-condensation derivative (*Z*)-**1c**, retaining one unreacted aldehyde function, was isolated in lower yield. The formation of this intermediate is consistent with the stepwise nature of the Knoevenagel condensation and suggests that the second condensation proceeds more slowly than the first under the adopted reaction conditions. Consistent with the stereochemical outcome generally observed for aurone-forming Knoevenagel condensations, both isolated products were assigned the (*Z*) configuration [12]. The symmetrical bis-aurone was therefore identified as the (Z,Z’)-isomer on the basis of its spectroscopic data and by comparison with closely related aurone derivatives previously reported in the literature [13].

For clarity, the structures of (*Z*)-**1c** and (*Z*,*Z*′)-**1a** shown in Scheme 1 are arbitrarily depicted in one of the possible conformations accessible through rotation about the relevant single bonds. In particular, rotation about the central 1,1′-biaryl bond of the biphenyl framework can generate different spatial arrangements of the two aromatic rings and, consequently, of the appended aurone moieties. Although such restricted biaryl rotation is formally related to atropisomeric behaviour, the structures shown here are intended only as conventional representations of molecular connectivity and configuration and do not imply the assignment of a specific persistent atropisomer or preferred solution conformation. The conformational features of (*Z*,*Z*′)-**1a** are discussed separately on the basis of the spectroscopic and computational results.

### 2.2. Photoisomerization Studies

The photoisomerization experiments were primarily aimed at generating new E-configured aurone derivatives from their corresponding Z isomers, thereby enabling their structural characterization and comparative analysis by NMR spectroscopy.

They were not designed to determine quantum yields, fatigue resistance, or intrinsic photoswitch performance. Aurone photochemistry can depend on substitution and molecular structure [14]; the observations reported here are restricted to the employed irradiation conditions.

The photoisomerization behaviour of both aurones, (*Z*)-**1c** and (*Z*,*Z’*)-**1a**, was investigated by ^1^H NMR spectroscopy in deuterated acetone under 254 nm irradiation. The choice of conditions was guided by our previous experiments on related α,β-unsaturated carbonyl compounds [15], rather than by a systematic wavelength or solvent screen of the present aurones.

Compound (*Z*)-**1c** underwent *Z*/*E* photoisomerization, giving an approximate (*Z*)/(*E*) ratio of 38/62 after 90 min of irradiation under the employed conditions (Figure 2). The corresponding (*E*)-**1c** isomer was isolated chromatographically and characterized by NMR spectroscopy and the analytical methods reported below. These observations allowed comparison of the structural and spectroscopic consequences of isomerization.

In contrast, the photochemical response of the bis-aurone (*Z*,*Z*′)-**1a** upon irradiation was considerably more complex. Instead of affording a single photoisomer, irradiation yielded an irradiated mixture consisting of the starting (*Z*,*Z*′)-**1a**, the mono-isomerized (*E*,*Z*)-**1a**, and trace amounts of an additional species tentatively attributed to the (*E*,*E*′)-**1a** isomer. Because the corresponding signals were very weak, this assignment could not be considered unambiguous. Only (*E*,*Z*)-**1a** could be isolated in pure form, whereas isolation and full characterization of the putative (*E*,*E*′)-**1a** isomer were not achieved (Figure 3).

Irradiation initially generated the mono-isomerized (*E*,*Z*)-**1a** species, while prolonged exposure produced weak additional signals possibly corresponding to (*E*,*E*′)-**1a**. A sequential single-unit isomerization pathway is consistent with these observations, but the identity of the minor component and the mechanism are not established. Its low abundance could reflect differences in the second isomerization step, absorption or excited-state properties, conformational effects, or the irradiation geometry at high concentration. The present data do not distinguish these possibilities. At 50 mM in an NMR tube, substantial inner-filter effects are expected; the observed compositions therefore depend on the experimental setup. Absorbance at 254 nm, replicate-derived standard deviations, and quantum yields were not determined. Deuterated acetone absorbs in the deep-UV region, and solvent-mediated photochemistry cannot be excluded. No evident major decomposition products were detected by the monitored ^1^H NMR spectra or TLC, although this does not exclude trace products.

### 2.3. Thermal Stability of (Z,Z′)-**1a** and (E,Z)-**1a**

Thermal stability experiments were performed in the dark on solutions of (*Z*,*Z*′)-**1a** and (*E*,*Z*)-**1a** in CDCl_3_ at 25 °C and 60 °C, monitored by ^1^H NMR spectroscopy. After 12 h at 60 °C, more than 99% of (*Z*,*Z*′)-**1a** remained, with no evident major decomposition (Scheme 2A).

On storage in the dark, (*E*,*Z*)-**1a** showed back-isomerization toward (*Z*,*Z*′)-**1a** without evident chemical degradation (Scheme 2B). These experiments provide qualitative evidence of thermal back-isomerization in solution, but do not allow the determination of rate constants, half-lives, or quantitative conversion–time profiles. Isolation of the photoisomer and its subsequent relaxation in solution describe different experimental stages and do not establish its solid-state persistence. Corresponding dark-relaxation data for (*E*)-**1c** are not available, so a comparative steric-stability argument is not made. No specific twisting, inversion, or catalytic mechanism is assigned.

### 2.4. Spectroscopic Studies

The structure of (*Z*,*Z*′)-**1a** was established by combining one-dimensional (^1^H and ^13^C) and two-dimensional (NOESY) NMR experiments, which allowed assignment of the diagnostic proton resonances and provided additional experimental evidence consistent with the assigned (Z,Z′) configuration. The NOESY spectrum of (*Z*,*Z*′)-**1a** (Figure 4) also provided information on the spatial relationships between selected regions of the molecule. In particular, diagnostic cross-peaks were observed between a biphenyl aromatic proton and both the olefinic proton of the aurone unit and a neighbouring methoxy group. These correlations enabled the olefinic resonance to be distinguished from the two aromatic proton signals of the 5,6-dimethoxybenzofuran-3(2H)-one moiety, which displayed closely similar chemical shifts and the same singlet multiplicity in the ^1^H NMR spectrum.

The observed through-space correlations are consistent with close spatial relationships between the corresponding nuclei but do not establish a unique solution conformation. The proposed (Z,Z′) configuration is further supported by comparison with structurally related aurones [12,13]. No single-crystal X-ray structure is available.

Additional NOESY correlations were observed between each of the two aromatic singlets of the 5,6-dimethoxybenzofuranone moiety and neighbouring methoxy protons. These interactions are consistent with the short interproton distances (approximately 2.34 Å for both pairs) found in the optimized geometry. However, because both aromatic protons exhibit analogous spatial relationships with their respective methoxy groups, these correlations do not permit an unambiguous assignment of their individual chemical shifts. Accordingly, the alternative assignments H4–H3 or H1–H2 are indicated in Figure 4.

Attention was subsequently focused on comparison of the ^1^H NMR spectra of (*Z*)-**1c** and (*Z*,*Z*′)-**1a** with those of their corresponding photogenerated (*E*)-**1c** and (*E*,*Z*)-**1a** derivatives (Figure 5). A particularly noteworthy spectroscopic feature was observed for both compounds containing an (*E*)-configured aurone unit: in each case, one aromatic proton belonging to the biphenyl moiety underwent a pronounced downfield shift, appearing at approximately δ 8.7–8.9 ppm. Such a high chemical shift is unusual for an aromatic proton in anisole or biphenyl structures. Similar strongly deshielded aromatic resonances have occasionally been reported for (*E*)-aurone derivatives, including photoresponsive aurones [8]; however, the structural origin of this unusual spectroscopic feature has not previously been examined in detail.

The occurrence of a similarly downfield-shifted biphenyl proton in both (*E*)-**1c** and (*E*,*Z*)-**1a** suggests that this spectroscopic anomaly may be associated with a related geometrical feature induced by the (*E*) configuration of the aurone unit. A possible contribution from the magnetic anisotropy of the carbonyl-containing conjugated system is examined computationally in Section 2.5. The spectra presented in Figure 5 were recorded in deuterated acetone, whereas the NOESY experiments were performed in CDCl_3_. Solvent-dependent conformational effects cannot therefore be excluded.

### 2.5. Computational Studies

Density Functional Theory (DFT) calculations [16,17] were performed to investigate the three-dimensional structure and conformational preferences of the title compound, to rationalize the unusual ^1^H NMR behaviour observed for the mono-photoisomerized (*E*,*Z*)-**1a** derivative, and to evaluate the structural consequences of photoinduced *Z*/*E* isomerization.

Geometry optimization of the parent (*Z*,*Z*′)-**1a** isomer revealed a markedly twisted biphenyl scaffold, in which the two aurone moieties adopt non-coplanar arrangements. Two representative conformations were considered, hereafter referred to as the compact (A) and more extended (C) conformers (Figure 6). The conformers differ primarily in the orientation of the aurone arms about their aryl–CH = single bonds rather than in the central biaryl dihedral. At B3LYP-D3(BJ)/def2-TZVP/SMD(chloroform)//B3LYP-D3(BJ)/def2-SVP/SMD(chloroform), ΔG(C − A) is +0.90 kcal mol^−1^ with conventional harmonic thermochemistry and +2.49 kcal mol^−1^ using a quasi-RRHO correction. The sensitivity of these relative energies to treatment of low-frequency modes precludes a firm conformer ranking.

This conformational comparison is particularly relevant to the NOESY data. Among the diagnostic through-space correlations observed for (*Z*,*Z*′)-**1a**, the interaction between a biphenyl aromatic proton and the olefinic proton is especially informative from a structural standpoint. The geometries shown in Figure 6 reveal short aromatic–olefinic H···H contacts of approximately 2.35 Å in both conformers A and C, although the aromatic proton involved differs between the two arrangements. These calculated proximities are consistent with the experimentally observed NOESY correlation but do not permit the unambiguous identification of a single conformer in solution.

Similarly, the observed correlation between a biphenyl aromatic proton and a neighbouring methoxy group provides additional evidence of local spatial proximity without establishing a unique molecular arrangement. Taken together, the experimental and computational results are consistent with different possible conformations of the (*Z*,*Z*′)-**1a** framework; the NOESY correlations do not establish their populations.

The same computational analysis was subsequently extended to the mono-photoisomerized (*E*,*Z*)-**1a** derivative. Optimization afforded two representative arrangements, conformers B and D (Figure 6). The calculated geometries indicate that conversion of one aurone unit from the Z to the E configuration modifies its local spatial relationship with the biphenyl moiety. For these conformers, ΔG(D − B) is −1.42 kcal mol^−1^ using conventional thermochemistry and +0.15 kcal mol^−1^ using quasi-RRHO at the def2-TZVP//def2-SVP level with SMD(chloroform). The sensitivity of the relative Gibbs free energies to the computational treatment, particularly for (*E*,*Z*)-**1a**, prevents an unambiguous assignment of the preferred solution conformer on energetic grounds alone (Table S1).

To further assess conformational mobility, relaxed scans were performed for rotation about the aryl–CH= bond of the aurone arms rather than the central biaryl bond. The scans indicate coupled structural relaxation and path dependence. For (*Z*,*Z*′)-**1a**, a transition-state optimization on one scan pathway yielded ΔG‡ = 11.3 kcal mol^−1^ at 298 K, corresponding formally to k ≈ 3.5 × 10^4^ s^−1^ from the Eyring equation (κ = 1). Reverse scans suggest a lower-energy pathway, but no transition state connecting its minima was fully characterized. Consequently, the lower barriers and associated rates should be regarded as estimates. The calculations are compatible with fast conformational exchange on the 600 MHz ^1^H NMR timescale but do not conclusively establish an interconversion pathway or rate.

Atropisomerization about the central biaryl bond was examined separately. A candidate transition-state structure displaying one imaginary frequency was located along the anti rotation pathway (biaryl dihedral ≈ 167°). At B3LYP-D3(BJ)/def2-SVP/SMD(chloroform), ΔG‡ = 10.3 kcal mol^−1^ relative to conformer A (10.9 kcal mol^−1^ with quasi-RRHO), corresponding to an Eyring rate estimate of approximately 2 × 10^5^ s^−1^ at 298 K for the conventional treatment. These results are consistent with atropisomeric interconversion at room temperature; the potentially lower-energy syn pathway was not fully characterized.

More importantly, photoisomerization produces a pronounced local reorganization around the biphenyl aromatic protons. In both optimized (*E*,*Z*)-**1a** conformers B and D, a diagnostic aromatic proton lies approximately 2.02–2.04 Å from the carbonyl oxygen of the E-aurone unit. Such arrangements may contribute to the unusual deshielding observed experimentally through effects associated with the local carbonyl-containing conjugated system, including magnetic anisotropy [18,19]. Geometrical proximity alone, however, cannot isolate the individual electronic contributions.

A similar geometrical effect may contribute to the unusually downfield methoxy resonance observed at δ 4.19 ppm in (*E*,*Z*)-**1a**. The calculated distance between the carbonyl oxygen and a methoxy proton decreases from 5.23 Å in the Z configuration to 3.85 Å in the E configuration, suggesting a possible contribution from the magnetic anisotropy of the carbonyl-containing conjugated system.

To examine this effect further, GIAO ^1^H and ^13^C NMR chemical-shift calculations were performed on the optimized conformers using B3LYP/pcS-1/SMD(chloroform), with TMS referencing. For the diagnostic biphenyl protons of (*E*,*Z*)-**1a**, calculated shifts are δ 10.02 ppm in B and δ 10.15 ppm in D, compared with experimental δ 8.90 ppm in CDCl_3_. Both conformers predict pronounced E-associated deshielding, albeit overestimating the absolute shift by approximately 1.1–1.3 ppm. Other aromatic and methoxy shifts agree better with experiment for the more extended conformers. Although this comparison suggests a contribution from open arrangements, quantitative conformer populations remain sensitive to the computational model and spectral assignments. Thus, the GIAO results support a local geometrical/electronic contribution to the diagnostic resonance without requiring a folded conformation.

Taken together, the spectroscopic and computational observations support a relationship between photoinduced geometrical isomerization and the local magnetic environment of selected aromatic protons in this biphenyl-bridged bis-aurone system; they do not establish a single preferred solution conformation.

## 3. Materials and Methods

### 3.1. General

Reagents were obtained from Sigma-Aldrich (Munich, Germany) and used without further purification. 1H and 13C NMR spectra were recorded in CDCl_3_ or acetone-d_6_ at 600 and 150 MHz, respectively, using a Bruker Avance III HD 600 MHz spectrometer (Palo Alto, CA, USA). Chemical shifts are reported in ppm (δ), and multiplicities are indicated as s (singlet) and d (doublet). Elemental analyses were performed using a PerkinElmer 240 C elemental analyzer (Waltham, MA, USA). Flash chromatography was performed on silica gel 60 (230–400 mesh; VWR, Radnor, PA, USA) using the stated solvent mixtures in v/v proportions. Photoisomerization experiments were carried out using a Spectroline ENF-240C/FE UV lamp operating at 254 nm (Fisher Scientific, Vantaa, Finland). Reactions were monitored by 1H NMR spectroscopy and analytical thin-layer chromatography (TLC) using 0.25 mm silica gel plates (60 F254; Sigma-Aldrich, Munich, Germany). Melting points were determined using a Büchi B-530 melting-point apparatus (Büchi, Flawil, Switzerland) and are uncorrected. Purity was assessed qualitatively by inspection of the ^1^H NMR spectra; a validated quantitative purity percentage or detection limit was not determined. ESI-HRMS spectra were acquired in positive-ion mode using a Thermo Scientific Q Exactive mass spectrometer. Molecular geometries, interatomic distances, and dihedral angles were analysed and rendered using UCSF Chimera (1.17.3) [20], whereas GaussView was used for structure inspection and analysis of the conformational energy profiles. Density functional theory (DFT) calculations were performed using Gaussian 16, Revision C.01 [21]. The four conformers were optimized at B3LYP-D3(BJ)/def2-SVP with SMD(chloroform) and characterized by harmonic frequencies (no imaginary frequencies). Free energies were evaluated at 298.15 K and 1 atm, with conventional and 100 cm^−1^ quasi-RRHO treatments and def2-TZVP single-point energies. GIAO shieldings were computed using B3LYP/pcS-1/SMD(chloroform) and referenced to consistently calculated TMS (σH = 31.279 ppm; σC = 181.137 ppm). Rotational profiles were assessed by forward and reverse relaxed scans at B3LYP-D3(BJ)/def2-SVP/SMD(chloroform).

### 3.2. Synthesis of (Z,Z’)-**1a** and (Z)-**1c**

Neutral alumina (10 equiv.) was added to a solution of the bisvanillin derivative **2** [22] (1 equiv.) and 5,6-dimethoxybenzofuran-3(2H)-one **3** [23] (2.2 equiv.) in the minimum volume of dichloromethane required for complete dissolution at room temperature. The reaction mixture was stirred at room temperature for 12 h. Upon completion of the reaction, the products were extracted from the alumina with dichloromethane. The combined organic extracts were filtered, and the solvent was removed under reduced pressure. The crude product was purified by flash chromatography using ethyl acetate/petroleum ether (1:2) as the eluent to afford the double-condensation product, the C_2_-symmetric bis-aurone (*Z*,*Z*′)-**1a**, (Rf = 0.35) in 60% isolated yield, together with the corresponding mono-condensation product (*Z*)-**1c**, (Rf = 0.40) which was isolated in 22% yield.

(*Z*)-**1c** (22%): mp 186–187 °C; ^1^H NMR (CDCl_3_) δ 3.71 (s, 3H), 3.78 (s, 3H), 3.89 (s, 3H), 3.98 (s, 3H), 3.99 (s, 6H), 6.75 (s, Ar, ^1^H), 6.78 (s, ^1^H), 7.16 (s, Ar, ^1^H), 7.41 (s, Ar, ^1^H), 7.43 (s, Ar, ^1^H), 7.47 (s, Ar, ^1^H), 7.50 (s, Ar, ^1^H), 9.99 (s, ^1^H); ^13^C NMR (CDCl_3_) δ 56.0, 56.4, 56.7, 57.0, 60.9, 60.9, 96.0, 103.8, 109.8, 111.7, 113.0, 115.2, 126.4, 128.0, 131.9, 132.0, 132.4, 146.6, 147.5, 148.4, 151.3, 152.5, 153.4, 157.6, 158.2, 163.2, 182.1, 190.9; Anal. Calcd. for C_28_H_26_O_9_: C, 66.40; H, 5.17. Found: C, 66.45; H, 5.26; ESI-HRMS calcd for C_28_H_26_O_9_ [M + H]^+^ 507.16496; found 507.16351. Rf = 0.40.

(*Z*,*Z*’)-**1a**: (60%): mp 261–262 °C; ^1^H NMR (CDCl_3_) δ 3.75 (s, 6H), 3.90 (s, 6H), 3.97 (s, 6H), 3.99 (s, 6H), 6.76 (s, 2H), 6.82 (s, Ar 2H), 7.17 (s, 2H), 7.48 (s, Ar, 2H), 7.49 (s, Ar, 2H); ^13^C NMR (CDCl_3_) δ 56.0, 56.4, 56.7, 60.8, 95.7, 104.0, 112.0, 113.0, 114.7, 126.7, 127.8, 132.7, 146.6, 147.5, 148.5, 152.7, 157.6, 163.6, 183.3; Anal. Calcd. for C_38_H_34_O_12_: C, 66.86; H, 5.02. Found: C, 66.66; H, 5.06; ESI-HRMS calcd for C_38_H_34_O_12_ [M + H]^+^ 683.21230; found 683.21003. Rf = 0.35.

### 3.3. Photoisomerization Monitored by ^1^H NMR of Compounds (Z)-**1c** and (Z,Z’)-**1a**

A 5 mm NMR tube containing a 50 mM solution of (*Z*)*-***1c** or (*Z*,*Z*′)*-***1a** in deuterated acetone was placed between two Spectroline UV lamps operating at 254 nm. The tube–lamp distance was approximately 2 cm and the temperature was approximately 20–22 °C. The tube was periodically mixed during irradiation. ^1^H NMR spectra were acquired at 0, 5, 10, 20, 60, and 180 min for (*Z*,*Z*′)*-***1a** and at 0, 15, 25, 45, 60, 90, and 120 min for (*Z*)*-***1c**. Approximate major-isomer ratios were obtained by integration of diagnostic biphenyl aromatic proton signals, normalized for the number of contributing protons. The weak minor component of the bis-aurone mixture was not quantified or included in the denominator. Absorbance and replicate-derived integration uncertainty were not determined. NMR and TLC monitoring revealed no evident major decomposition products. Photoisomer-enriched mixtures were purified by flash chromatography (ethyl acetate/petroleum ether, 1:2, v/v), affordin*g* (*E*)-**1c** (Rf = 0.33) and (*E*,*Z*)*-***1a** (Rf = 0.30) as yellow solids. The isolated yields are calculated from moles of purified photoisomer relative to initial moles of the corresponding Z starting isomer, rather than relative to its estimated amount in the irradiated mixture.

(*E*)-**1c** (58%): mp 145–146 °C; 1H NMR (CDCl3) δ 3.71 (s, 3H), 3.81 (s, 3H), 3.89 (s, 3H), 3.98 (s, 3H), 4.02 (s, 3H), 4.04 (s, 3H), 6.93 (s, Ar, 1H), 7.03 (s, Ar, 1H), 7.15 (s, Ar, 1H), 7.47 (s, Ar, 1H), 7.54 (s, Ar, 1H), 7.60 (s, Ar, 1H), 8.69 (s, Ar, 1H), 9.99 (s, 1H); 13C NMR (CDCl3) δ 55.5, 55.5, 55.7, 56.0, 60.1, 60.4, 95.3, 112.5, 114.5, 115.2, 119.6, 121.3, 122.1, 123.3, 125.1, 125.7, 127.8, 131.7, 133.0, 146.6, 146.7, 148.7, 152.4, 153.8, 157.1, 162.5, 180.8, 192.5; Anal. Calcd. for C28H26O9: C, 66.40; H, 5.17. Found: C, 66.54; H, 5.13; ESI-HRMS calcd for C28H26O9 [M + H]^+^ 507.16496; found 507.16382. Rf = 0.33.

(*E*,*Z*)-**1a** (48%); mp 225–226 °C; 1H NMR (CDCl3) δ 3.82 (s, 3H), 3.86 (s, 3H), 3.94 (s, 3H), 3.96 (s, 3H), 4.03 (s, 3H), 4.06 (s, 3H), 4.09 (s, 3H), 4.19 (s, 3H), 6.72 (s, Ar, 1H), 6.87 (s, Ar, 1H), 6.89 (s, Ar, 1H), 6.89 (s, Ar, 1H), 7.21 (s, Ar, 1H), 7.24 (s, Ar, 1H), 7.29 (s, Ar, 1H), 7.52 (s, Ar, 1H), 7.58 (s, Ar, 1H), 8.90 (s, Ar, 1H); 13C NMR (CDCl3) δ 56.0, 56.0, 56.0, 56.6, 56.7, 56.8, 60.9, 60.9, 95.0, 95.7, 95.8, 103.7, 104.0, 112.0, 112.1, 114.8, 114.9, 122.6, 125.0, 126.8, 127.7, 127.9, 130.9, 130.9, 132.8, 135.2, 146.4, 146.7, 147.6, 148.5, 152.4, 152.8, 157.6, 157.9, 162.6, 163.4, 181.6, 183.9; Anal. Calcd. for C38H34O12: C, 66.86; H, 5.02. Found: C, 66.82; H, 5.00; ESI-HRMS calcd for C_38_H_34_O_12_ [M + H]^+^ 683.21230; found 683.21063. Rf = 0.30.

## 4. Conclusions

A highly oxygenated biphenyl-bridged bis-aurone and its mono-condensation derivative were synthesized and characterized by NMR spectroscopy and the analytical methods reported herein. The description of the parent framework as C_2_-symmetric denotes its constitutionally equivalent halves rather than an assigned rigid solution conformation.

Both derivatives underwent UV-induced *Z*/*E* isomerization under the employed NMR-tube conditions. The major *E*-containing photoisomers were isolated; weak additional signals in the bis-aurone mixture may correspond to (*E*,*E*′)-**1a**, but that assignment remains tentative. The reported compositions are approximate and do not establish intrinsic switching efficiency.

NOESY correlations establish short-range spatial contacts but do not identify a unique solution conformer; the calculated geometries of both compact and more extended arrangements can accommodate the diagnostic contacts.

The E-containing derivatives exhibit a strongly downfield biphenyl proton. GIAO calculations support a local geometric and electronic contribution associated with the E-aurone carbonyl region in both compact and extended conformers, without uniquely establishing the individual magnetic contributions.

The study connects the synthesis of a biphenyl-linked aurone framework with NMR observations of geometrical isomerization and its possible conformational consequences. Quantitative photophysics and thermal kinetics remain outside the experimentally established scope.

## Figures and Tables

**Figure 1:**
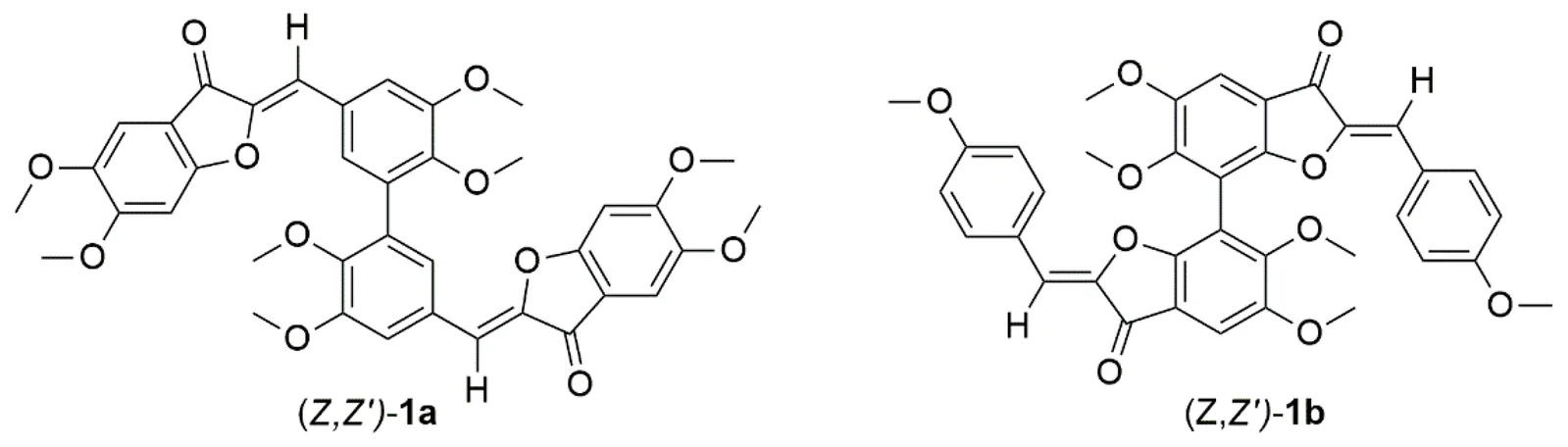
Chemical structures of compounds (*Z*,*Z*′)-**1a** and (*Z*,*Z*′)-**1b**. Both compounds are arbitrarily depicted in one of their possible conformations.

**Figure 2:**
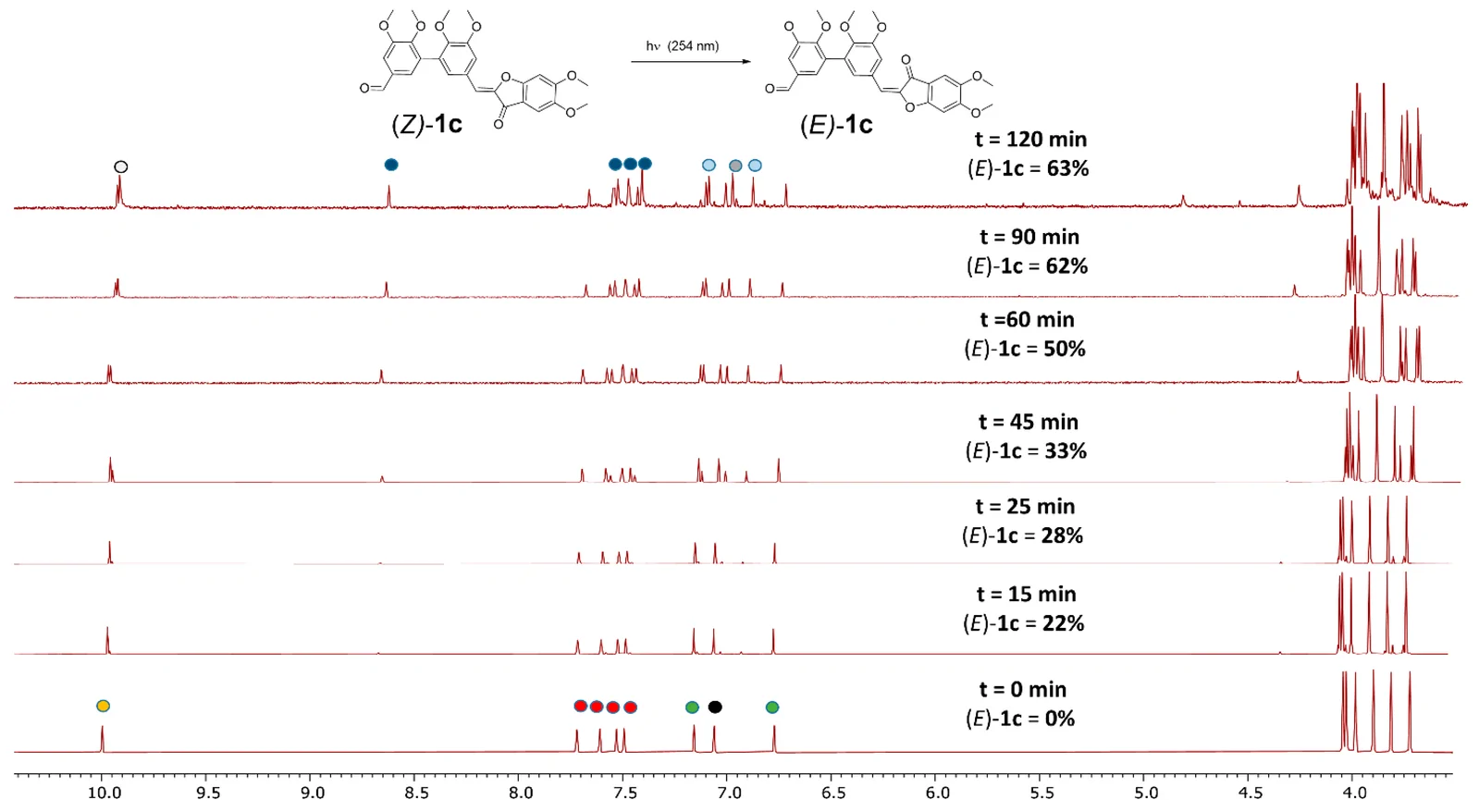
Time-dependent ^1^H NMR spectra recorded in deuterated acetone during irradiation of (*Z*)-**1c** at 254 nm for 0, 15, 25, 45, 60, 90, and 120 min, showing the progressive formation of (*E*)-**1c**. The reported percentages refer to the relative amount of the (*E*) isomer, calculated as [(*E*)-**1c**]/[(*E*)-**1c** + (*Z*)-**1c**] × 100 from integration of diagnostic biphenyl aromatic proton signals, with integrals normalized for the number of contributing protons. In the (*Z*) isomer (t = 0 min), the red circles indicate the four aromatic proton signals of the biphenyl moiety, the green circles highlight the two aromatic proton signals of the 5,6-dimethoxybenzofuran-3(2H)-one ring, the yellow circle marks the aldehydic proton signal, and the black circle identifies the olefinic proton. In the (*E*) isomer (t = 120 min), the blue circles indicate the four aromatic proton signals of the biphenyl moiety, the light blue circles highlight the two aromatic proton signals of the 5,6-dimethoxybenzofuran-3(2H)-one ring, the white circle marks the aldehydic proton signal, and the grey circle identifies the olefinic proton of the aurone moiety. These percentages are approximate compositions under the employed irradiation conditions; replicate-derived uncertainties were not determined.

**Figure 3:**
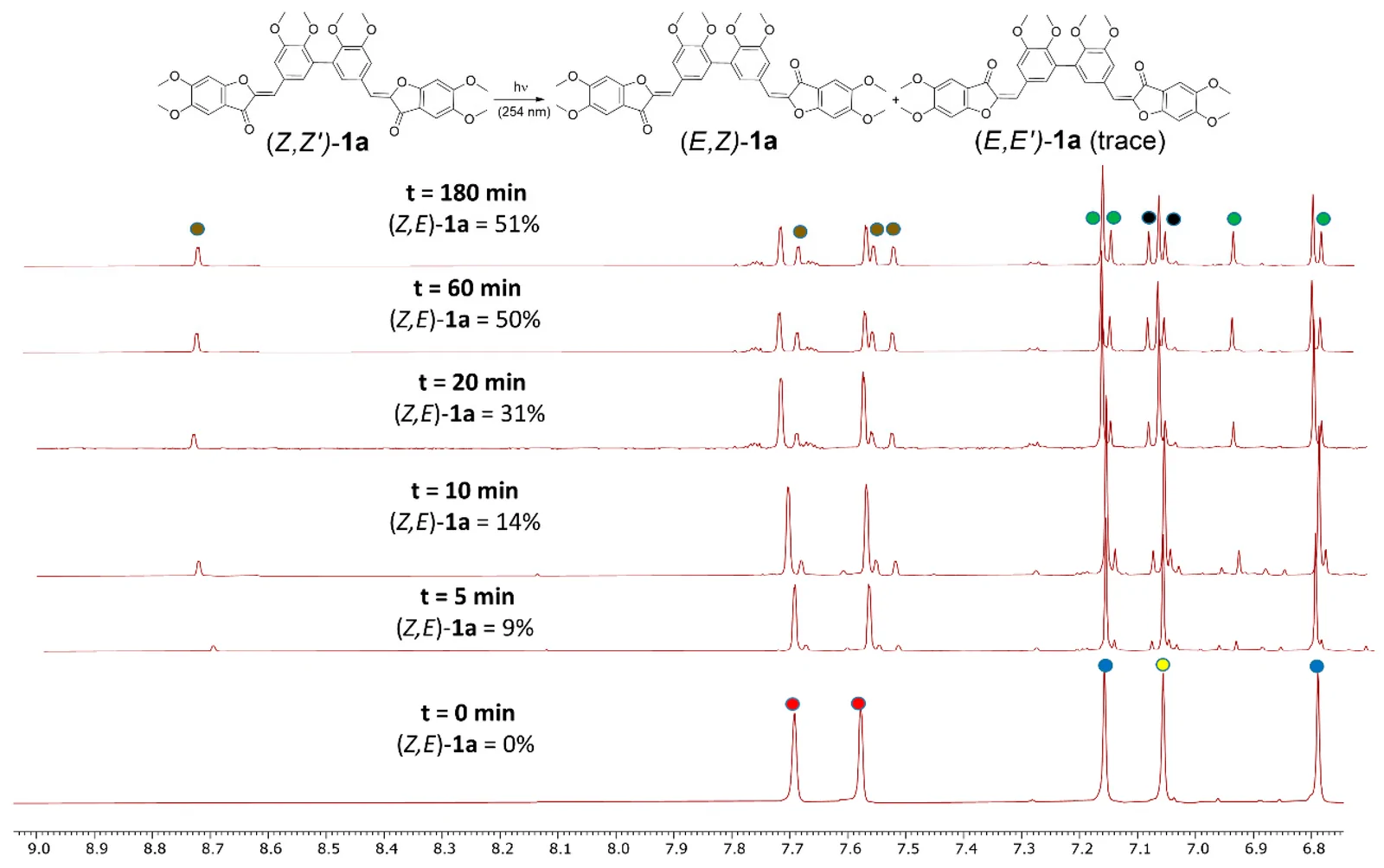
Time-dependent ^1^H NMR spectra recorded in deuterated acetone during irradiation of (*Z*,*Z*′)-**1a** at 254 nm for 0, 5, 10, 20, 60, and 180 min, showing the formation of (*E*,*Z*)-**1a**. The reported percentages refer to the relative amount of the (*E*,*Z*)-**1a** isomer, calculated as [(*E*,*Z*)-**1a**]/[(*E*,*Z*)-**1a** + (*Z*,*Z*′)-**1a**] × 100 from integration of diagnostic biphenyl aromatic proton signals, with integrals normalized for the number of contributing protons. In the ^1^H NMR of (*Z*,*Z*′)-**1a** isomer (t = 0 min), the red circles identify the two aromatic proton signals of the biphenyl moiety, the blue circles mark the aromatic proton signals of the 5,6-dimethoxybenzofuran-3(2H)-one rings, and the yellow circles indicate the olefinic protons. In the ^1^H NMR of (*E*,*Z*)-**1a** isomer (t = 180 min), the black circles indicate the olefinic protons of the aurone moieties, whereas the green and grey circles identify the aromatic proton signals of the 5,6-dimethoxybenzofuran-3(2H)-one rings and of the biphenyl moiety, respectively. These percentages are approximate compositions under the employed irradiation conditions; replicate-derived uncertainties were not determined. The ratio is normalized to the two major species and excludes the weak, unassigned minor component; it is not the fraction of all aurone units in the E configuration.

**Figure 4:**
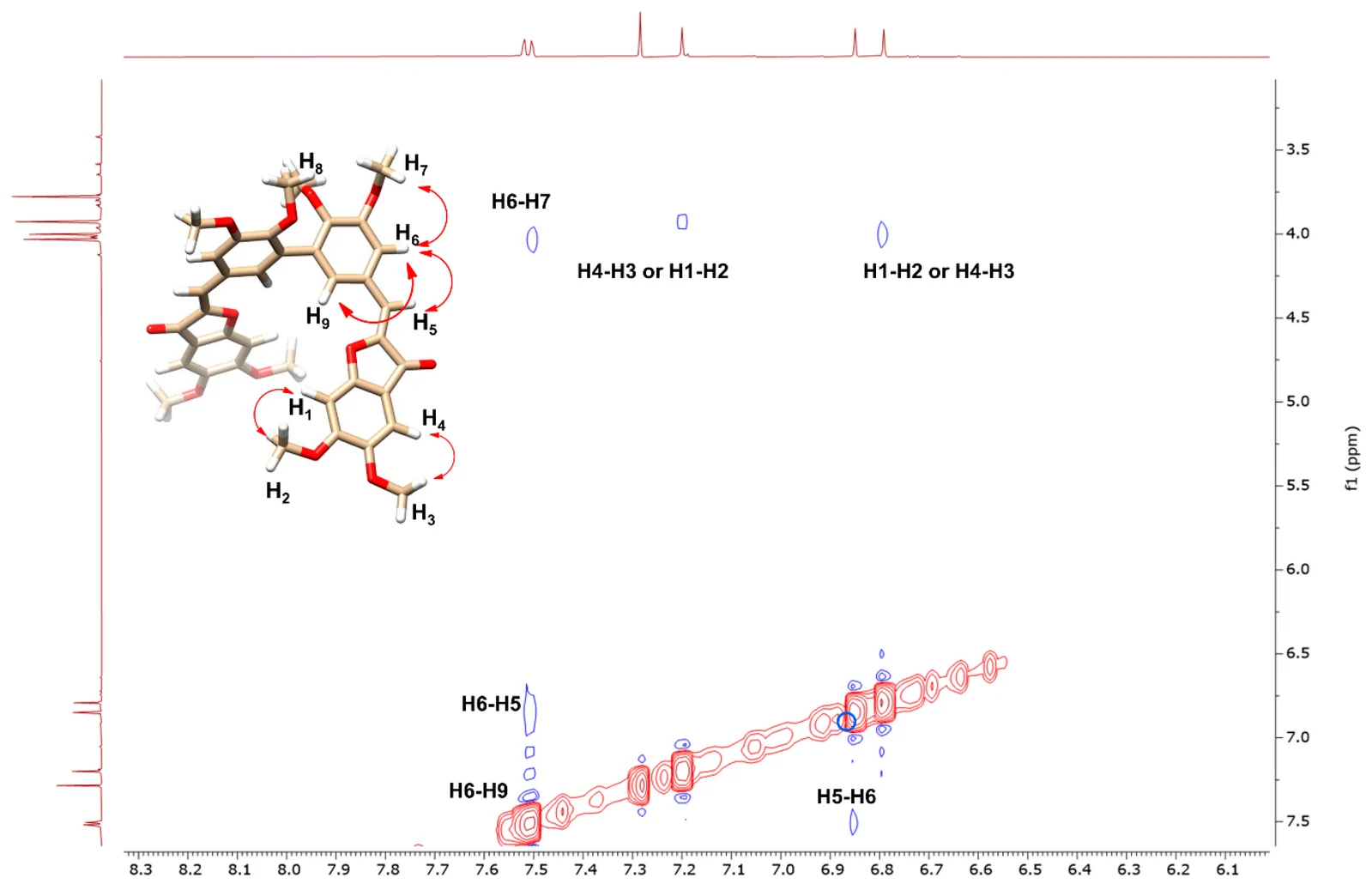
NOESY NMR spectrum of (*Z*,*Z*′)-**1a** recorded in CDCl_3_, showing the diagnostic through-space correlations used for proton assignment and conformational analysis. Cross-peaks between a biphenyl aromatic proton and both the olefinic proton and a neighbouring methoxy group are highlighted. Additional correlations involve the aromatic protons of the 5,6-dimethoxybenzofuranone moiety and their neighbouring methoxy groups. Alternative assignments are indicated where the individual aromatic resonances cannot be distinguished unambiguously.

**Figure 5:**
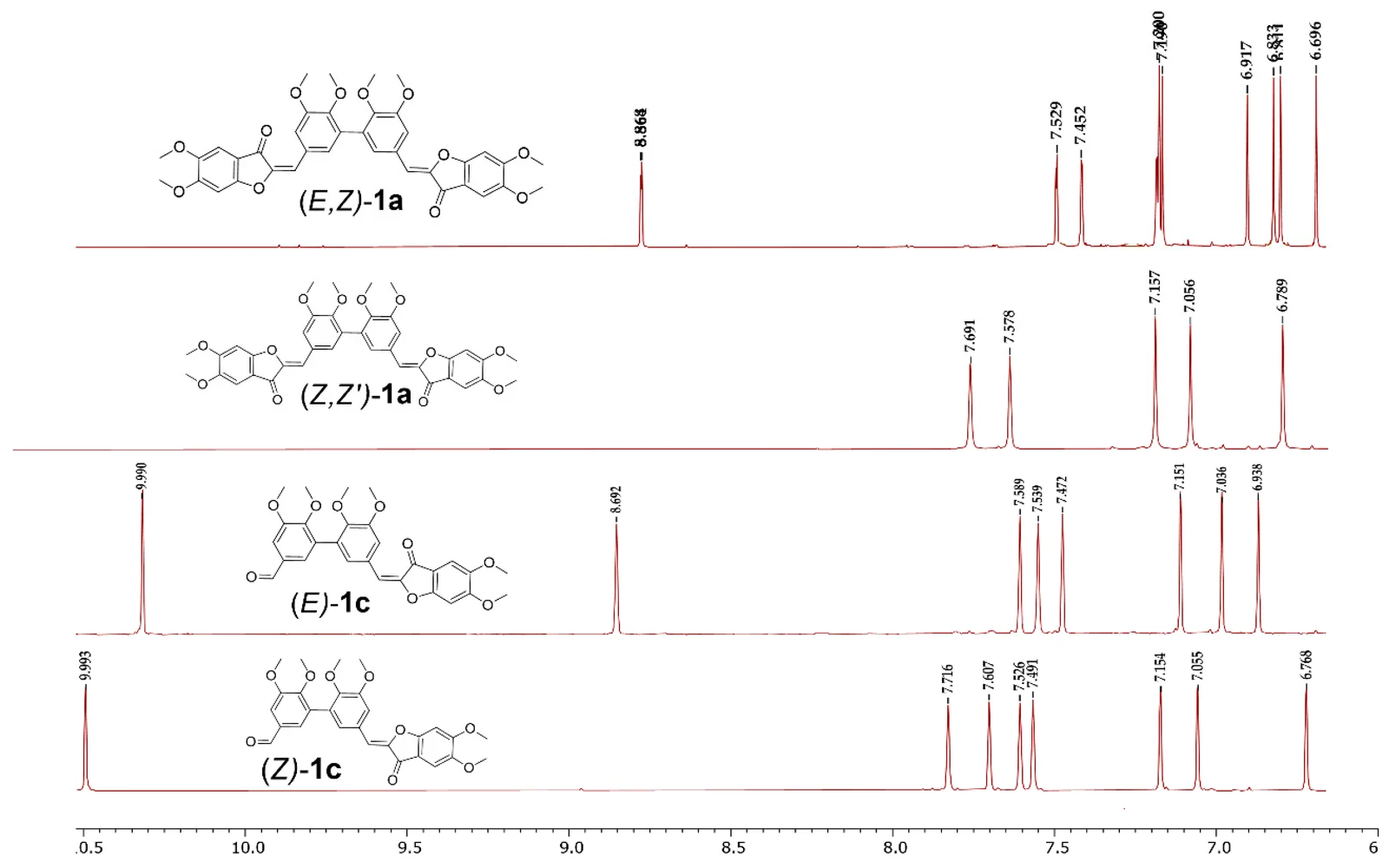
Comparison of the ^1^H NMR spectra (aromatic and olefinic regions) recorded in deuterated acetone of (*Z*)-**1c**, (*E*)-**1c**, (*Z*,*Z*′)-**1a**, and (*E*,*Z*)-**1a**.

**Figure 6:**
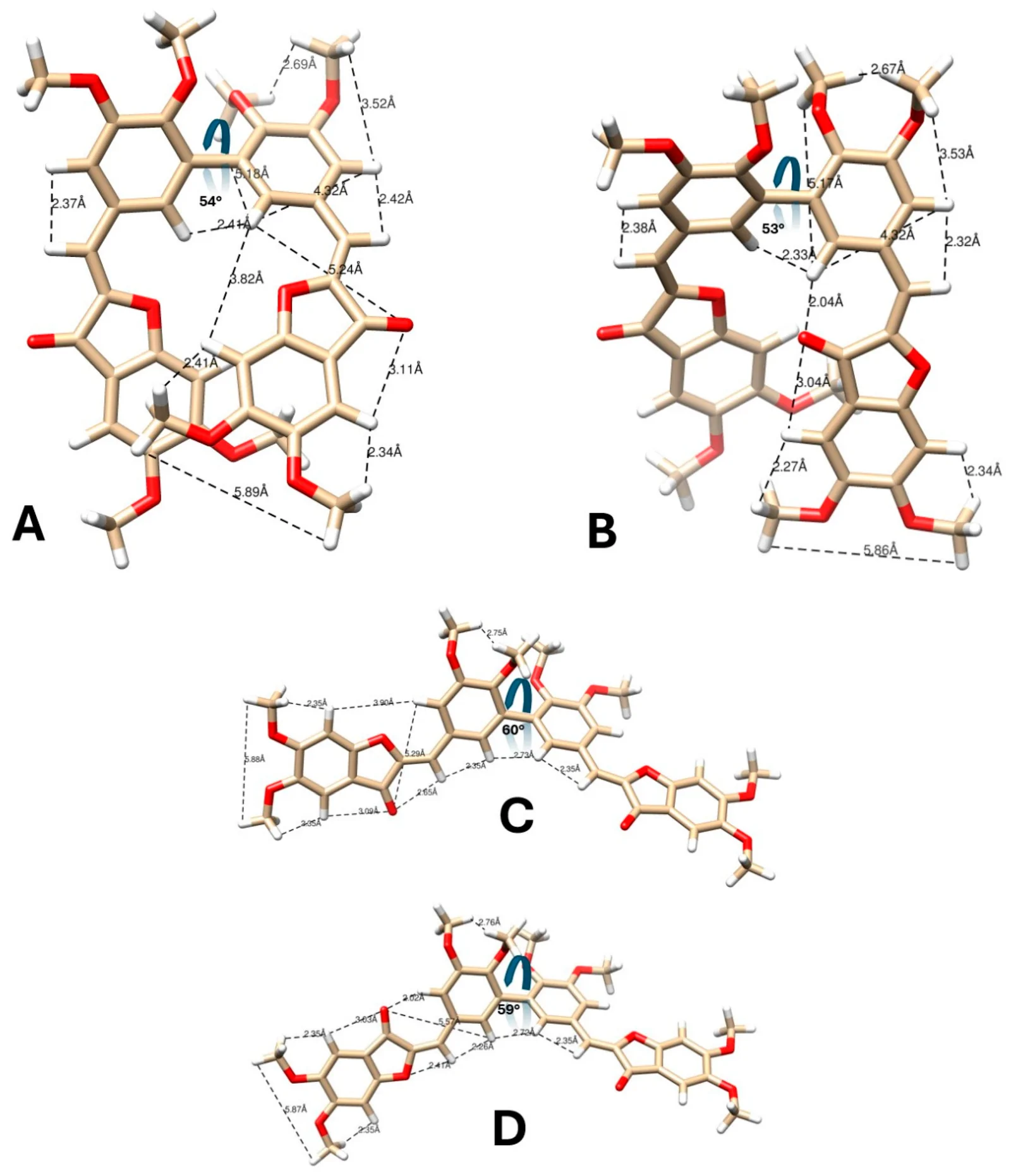
DFT-optimized representative conformations of (*Z*,*Z*′)-**1a** and (*E*,*Z*)-**1a**. (**A**) Compact conformer of (*Z*,*Z*′)-**1a**; (**B**) compact conformer of (*E*,*Z*)-**1a**; (**C**) more extended conformer of (*Z*,*Z*′)-**1a**; and (**D**) more extended conformer of (*E*,*Z*)-**1a**. Selected calculated intramolecular distances relevant to the NOESY correlations and aromatic deshielding, together with the central biphenyl dihedral angles, are indicated. The conformational labels refer to representative arrangements rather than rigid structural categories.

**Scheme 1:**
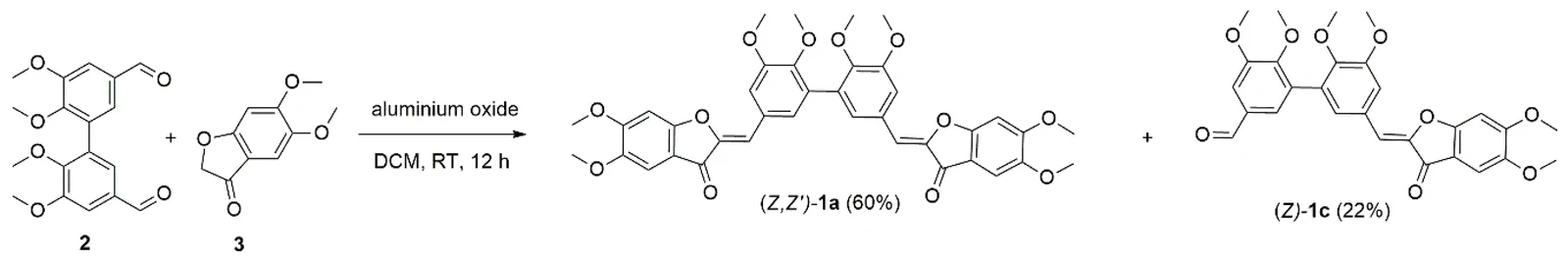
Synthesis of compounds (*Z*,*Z’*)-**1a** and (*Z*)-**1c** by Knoevenagel condensation reaction.

**Scheme 2:**
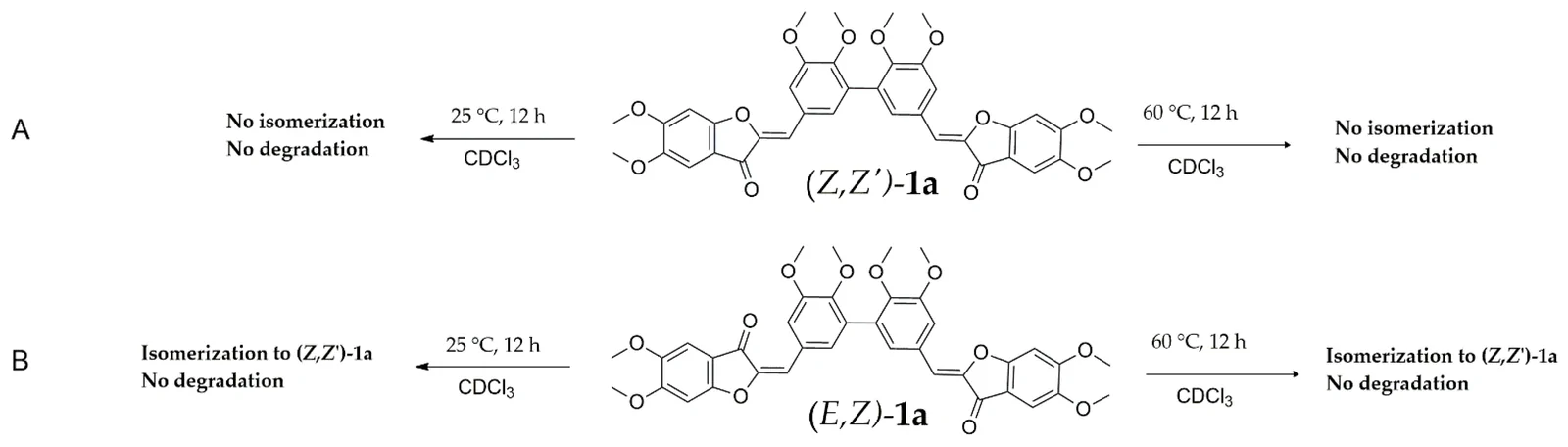
Thermal behaviour of (*Z*,*Z*′)-**1a** and (*E*,*Z*)-**1a** in CDCl_3_ at 25 and 60 °C under dark conditions. (**A**) Stability of (*Z*,*Z*′)-**1a**. (**B**) Back-isomerization of (*E*,*Z*)-**1a** to (*Z*,*Z*′)-**1a**. The arrows in (**B**) schematically represent the observed transformation without implying quantitative conversion or kinetic information.

## Data Availability

The spectroscopic data supporting this study are provided in the Supplementary Materials.

## Supplementary Materials

The following supporting information can be downloaded at: https://www.mdpi.com/article/10.3390/molecules31213963/s1. Figure S1: ^1^H NMR of compound (*Z*)-**1c**. Figure S2: ^13^C NMR of compound (*Z*)-**1c.** Figure S3: ^1^H NMR of compound (*E*)-**1c**. Figure S4: ^13^C NMR of compound (*E*)-**1c**. Figure S5: ^1^H NMR of compound (*Z*,*Z’*)-**1a**. Figure S6: ^13^C NMR of compound (*Z*,*Z’*)-**1a**. Figure S7: ^1^H NMR of compound (*E*,*Z*)-**1a**. Figure S8: ^13^C NMR of compound (*E*,*Z*)-**1a**. Figure S9: HRMS (ESI^+^) of compound (*Z*)-**1c**. Figure S10: HRMS (ESI^+^) of compound (*E*)-**1c**. Figure S11: HRMS (ESI^+^) of compound (*Z*,*Z’*)-**1a**. Figure S12: HRMS (ESI^+^) of compound (*E*,*Z*)-**1a**. Figure S13. GIAO-calculated ^1^H NMR chemical shifts and DFT-optimized geometry of the compact conformer B of (*E*,*Z*)-**1a**. The diagnostic biphenyl aromatic proton H_5_ (δ 10.02 ppm) is located 2.04 Å from the carbonyl oxygen of the E-aurone unit. Atom labels in this figure follow the numbering adopted for the GIAO calculations and do not correspond to the proton numbering used in Figure 4. Figure S14. GIAO-calculated ^1^H NMR chemical shifts and DFT-optimized geometry of the more extended conformer D of (*E*,*Z*)-**1a**. The diagnostic biphenyl aromatic proton H_8_ (δ 10.15 ppm) is located 2.02 Å from the carbonyl oxygen of the E-aurone unit. Atom labels in this figure follow the numbering adopted for the GIAO calculations and do not correspond to the proton numbering used in Figure 4. Table S1. Relative conformational energies of the investigated bis-aurones.
