# Supplementary material for: A C2-Symmetric Biphenyl-Bridged Bis-Aurone: Synthesis, Z/E Photoisomerization, and Conformational Insights from NMR Spectroscopy and DFT Analysis
Source: Molecules. 2026 Nov 3;31(21):3963. doi: 10.3390/molecules31213963 (PMC13765349; doi:10.3390/molecules31213963)
Supplement: Supplementary file 1 [file molecules-31-03963-s001.zip › molecules-4617928-supplementary.pdf]

Supplementary Materials for

A C_2-Symmetric Biphenyl-Bridged Bis-Aurone: Synthesis, *Z/E* Photoisomerization, and Conformational Insights from NMR Spectroscopy and DFT Analysis

Maria Antonietta Dettori, Davide Fabbri*, Roberto Dallocchio, Nicola Culeddu, Emanuela Azara and Paola Carta

Istituto di Chimica Biomolecolare, Consiglio Nazionale delle Ricerche, Traversa La Crucca 3, I–07100 Sassari, Italy.
* Correspondence: davidegaetano.fabbri@cnr.it

Table of contents

Copies of ^{1}H NMR and ^{13}C NMR spectra ($CDCl_3$) of compound (*Z*)-**1c.** **page 2**

Copies of ^{1}H NMR and ^{13}C NMR spectra ($CDCl_3$) of compound (*E*)-**1c................page 3**

Copies of ^{1}H NMR and ^{13}C NMR spectra ($CDCl_3$) of compound (*Z,Z′*)-**1a...........page 4**

Copies of ^{1}H NMR and ^{13}C NMR spectra ($CDCl_3$) of compound (*E,Z*)-**1a.............page 5**

Copies of HRMS (ESI^+) of compounds: (*Z*)-**1c**, (*E*)-**1c** and (*Z,Z′*)-**1a.** **page 6**

Copies of HRMS (ESI^+) of compound: (*E,Z*)-**1a.** **page 7**

Copies of optimized geometry of conformer B of compound *(E,Z)*-**1a**.

and corresponding calculated GIAO ^{1}H NMR chemical shifts. **page 7**

Copies of optimized geometry of conformer D of compound *(E,Z)*-**1a**.

and corresponding calculated GIAO ^{1}H NMR chemical shifts. **page 8**

Table S1. Relative conformational energies of the investigated bis-aurones. **page 8**

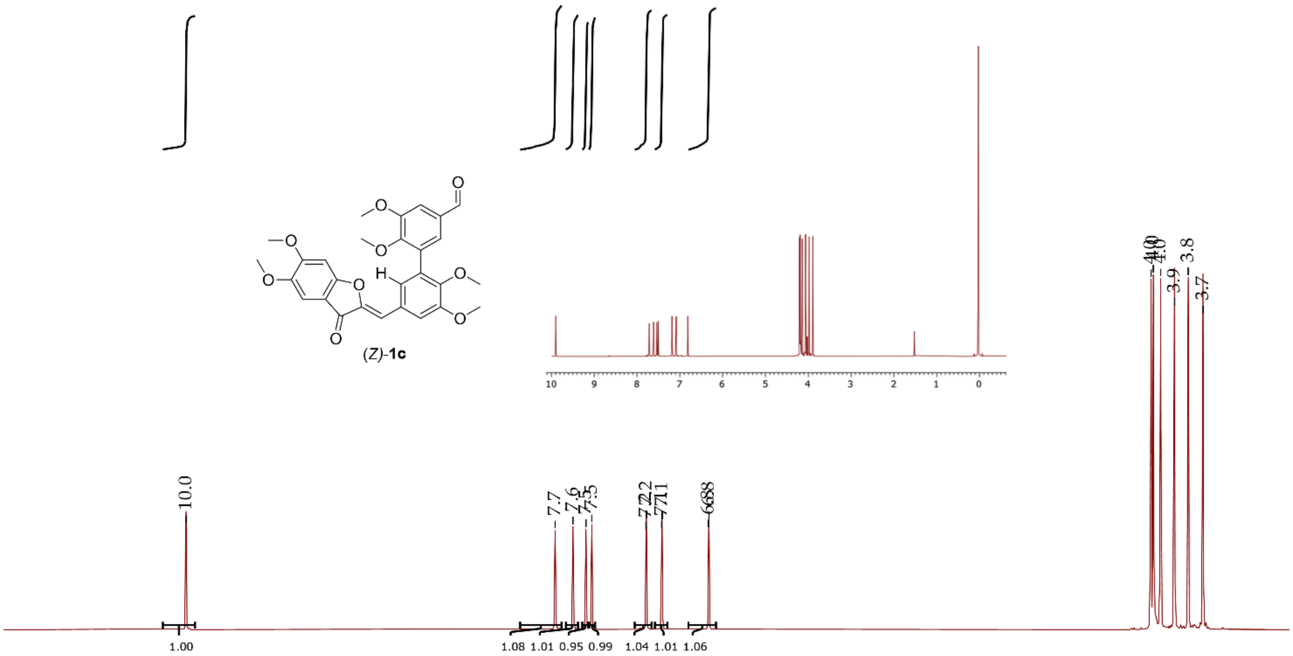

Figure S1. ^{1}H NMR spectrum of compound (Z)-1c.

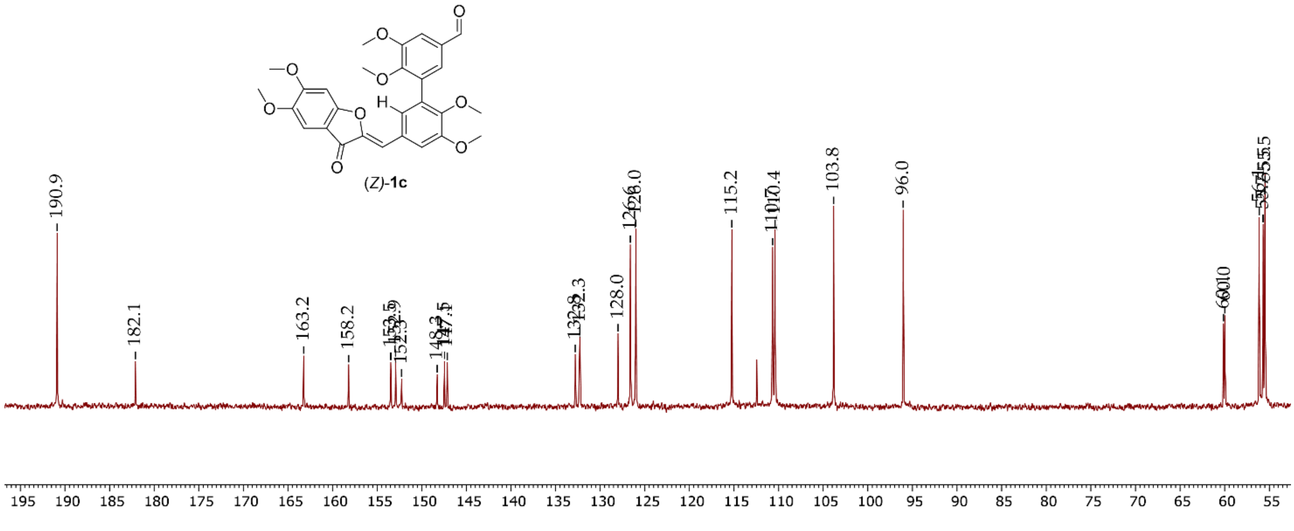

Figure S2. ^{13}C NMR spectrum of compound (Z)-1c.

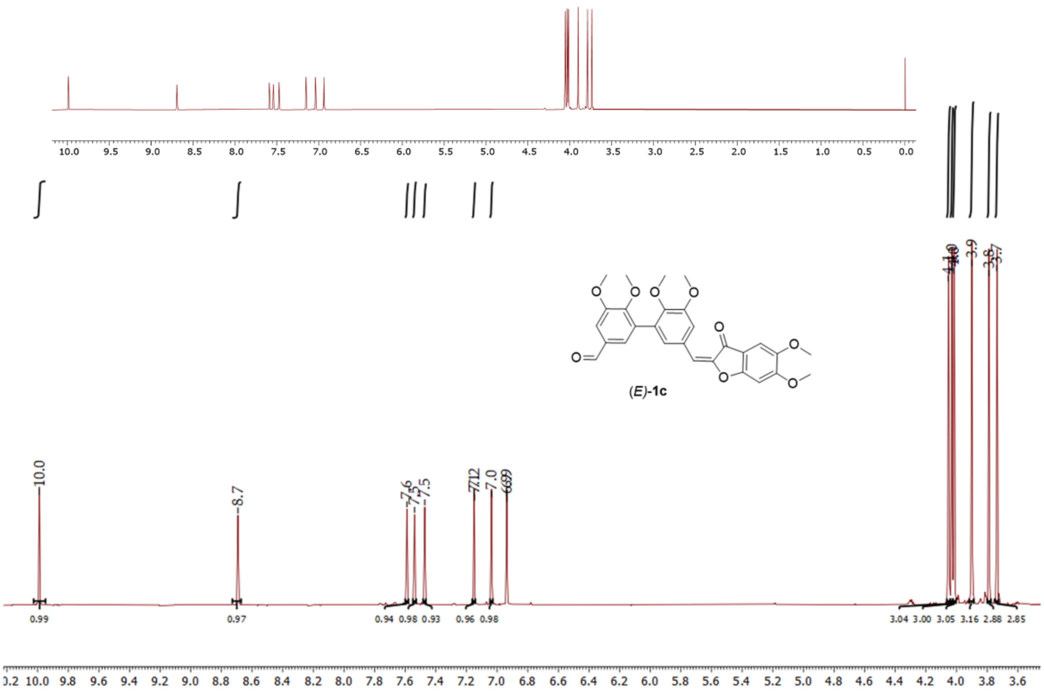

Figure S3. ^{1}H NMR spectrum of compound (E)-1c.

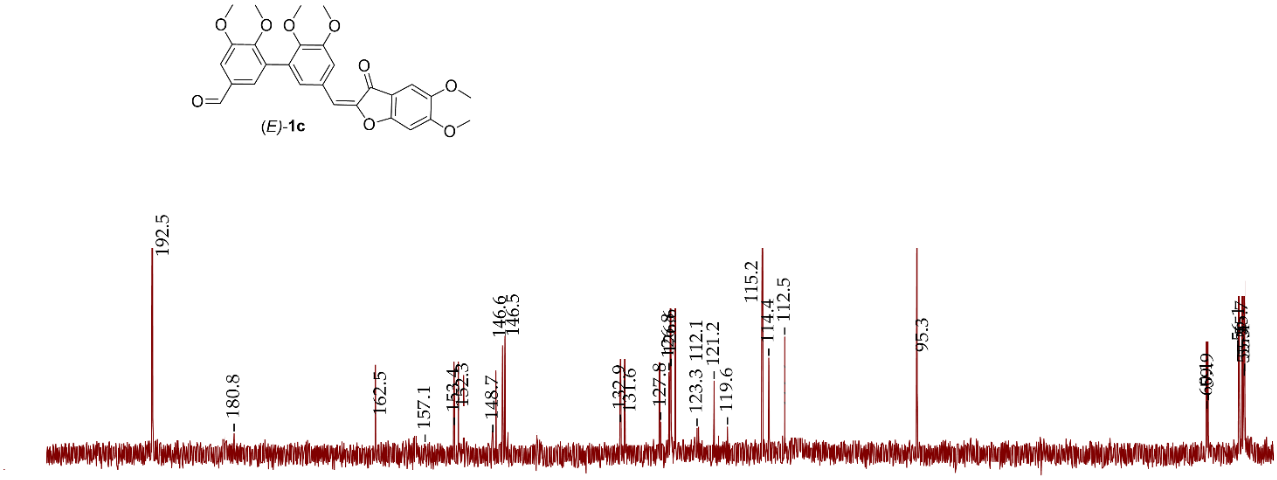

Figure S4. ^{13}C NMR spectrum of compound (E)-1c.

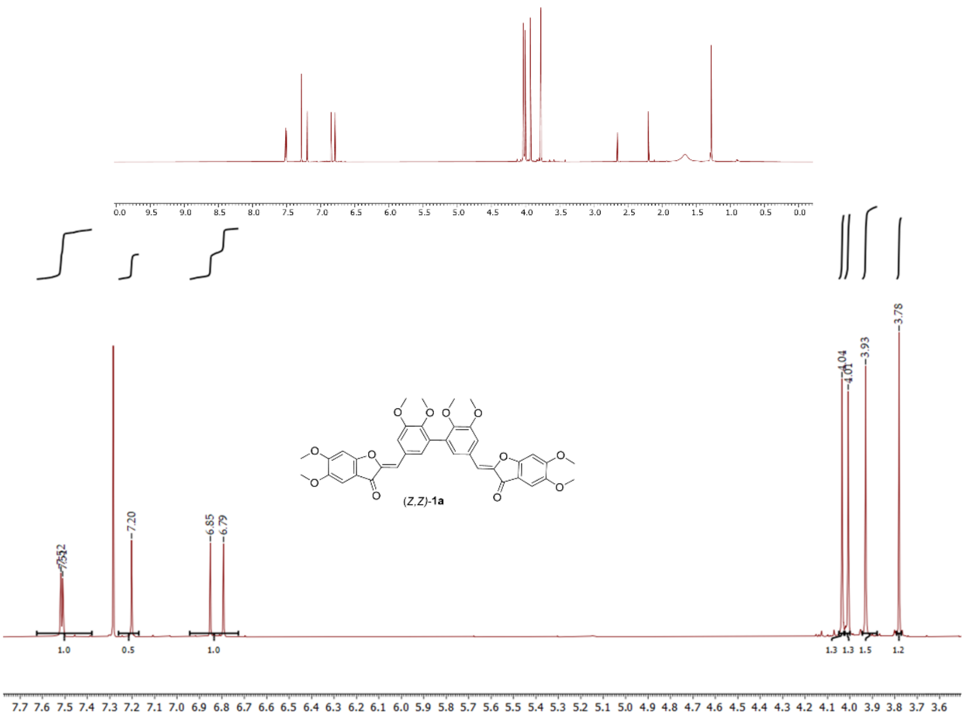

Figure S5. ¹H NMR spectrum of compound (Z,Z′)-1a.

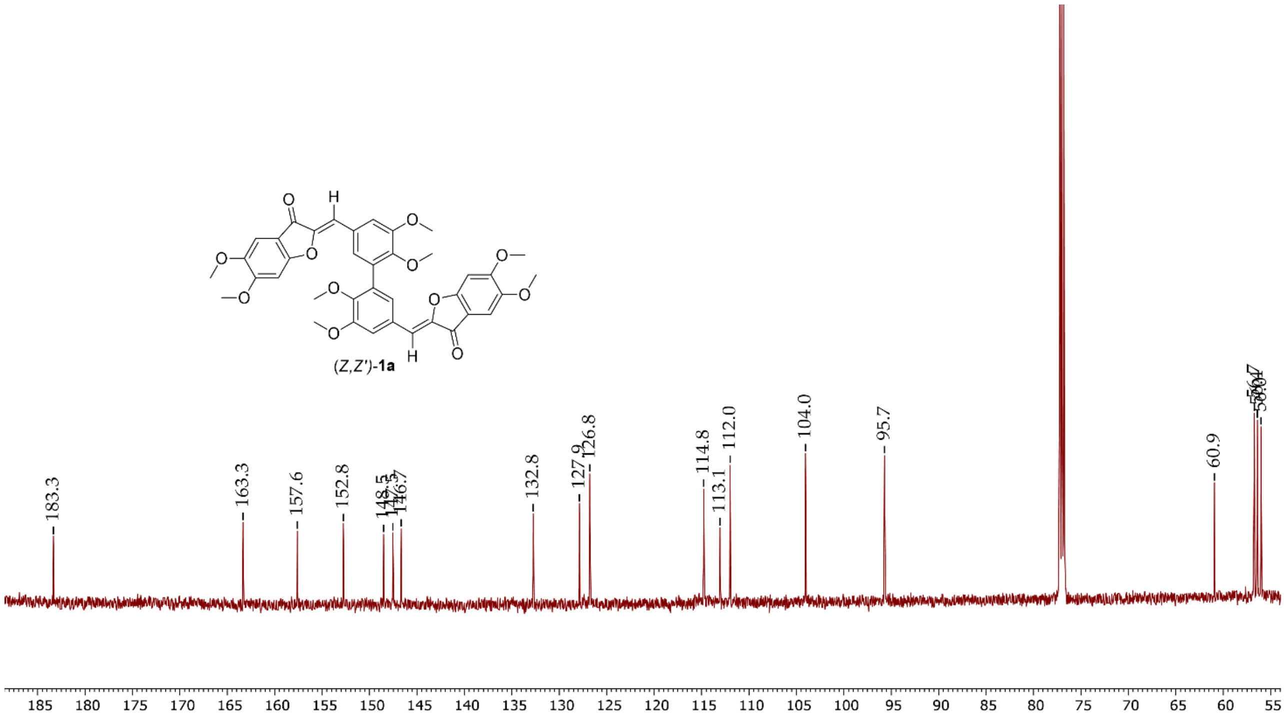

Figure S6. ¹³C NMR spectrum of compound (Z,Z′)-1a.

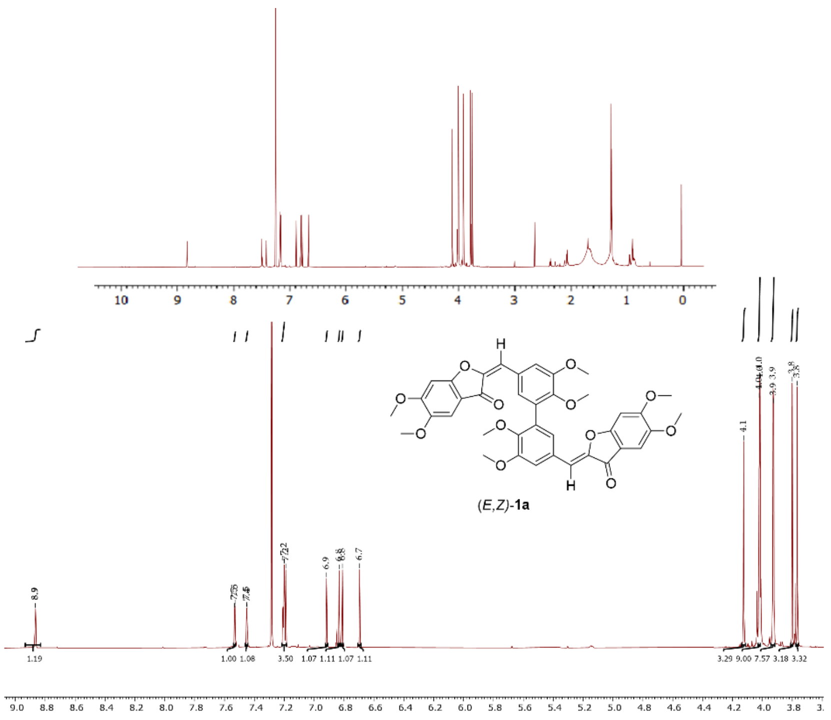

Figure S7. **^{1}H NMR spectrum of compound (E,Z)-1a.**

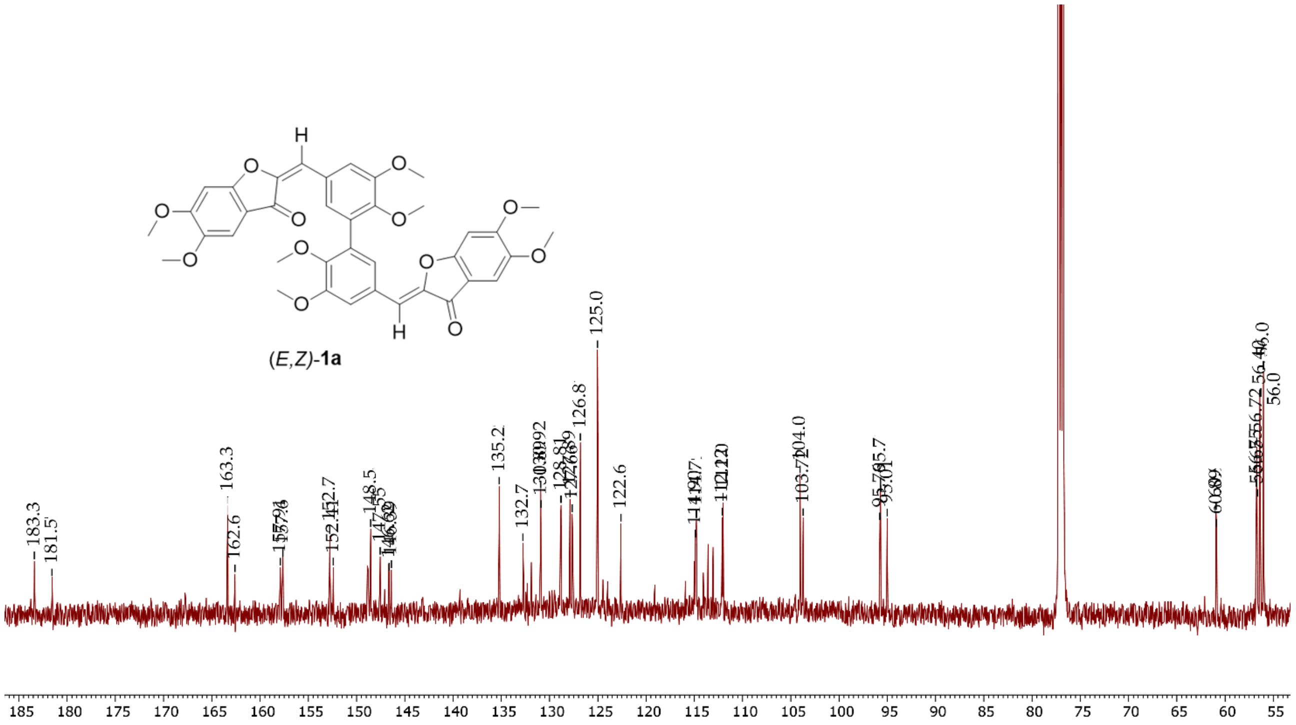

Figure S8. **^{13}C NMR spectrum of compound (E,Z)-1a.**

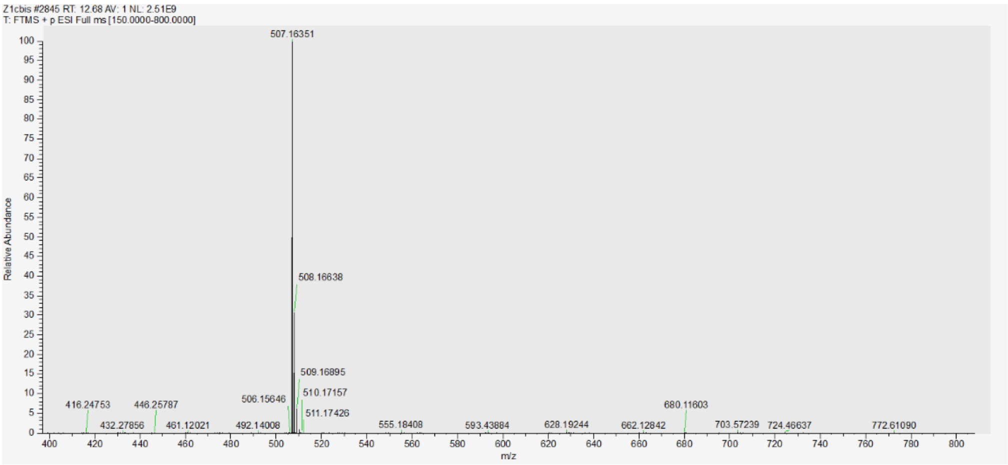

Figure S9. HRMS (ESI⁺) of compound (*Z*)-**1c.**

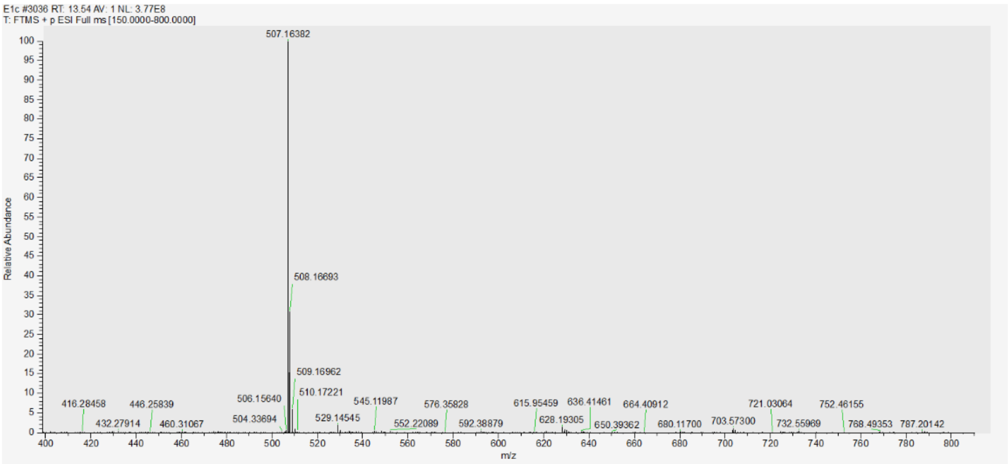

Figure S10. HRMS (ESI⁺) of compound (*E*)-**1c.**

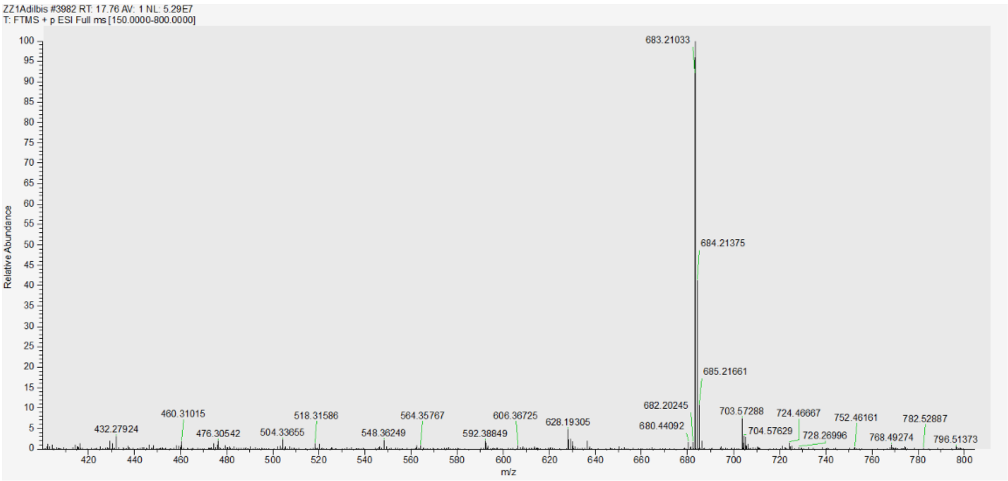

Figure S11. HRMS (ESI⁺) of compound (*Z*,*Z′*)-**1a.**

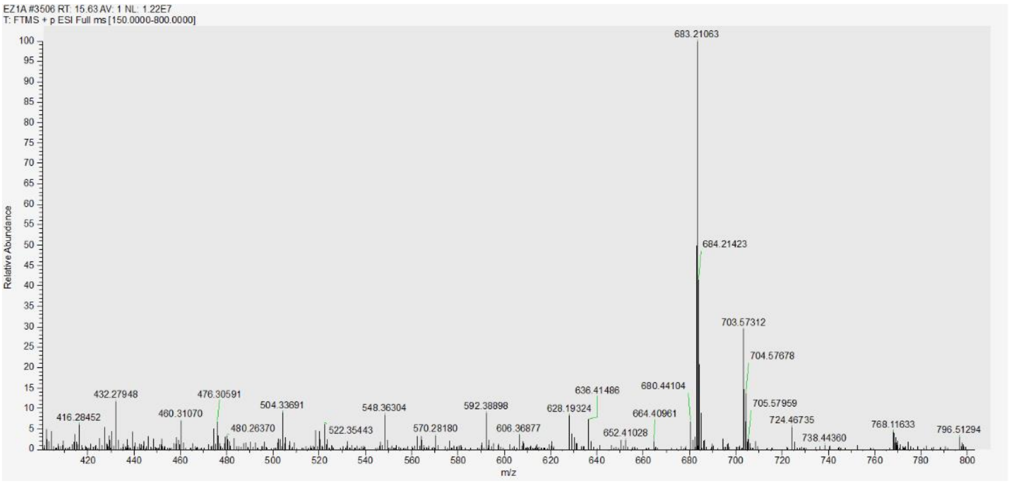

Figure S12. HRMS (ESI+) of compound (*E*,*Z*)-**1a.**

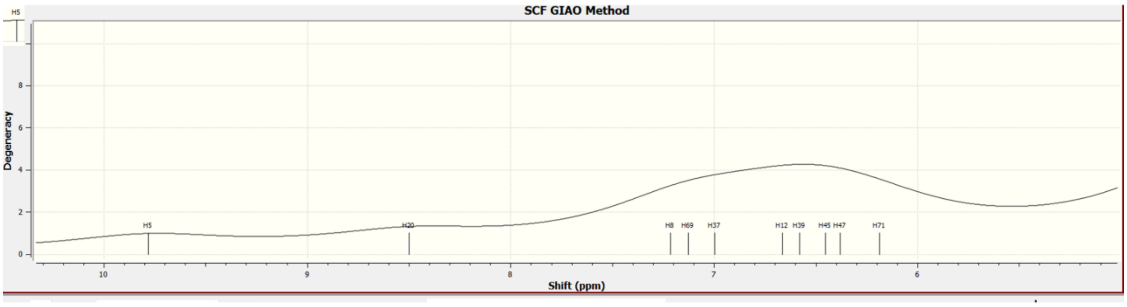

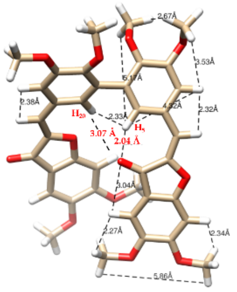

(*E*,*Z*)-**1a**

Figure S13.

GIAO-calculated ^{1}H NMR chemical shifts and DFT-optimized geometry of the compact conformer B of (*E*,*Z*)-**1a**. The diagnostic biphenyl aromatic proton H_5 (δ 10.02 ppm) is located 2.04 Å from the carbonyl oxygen of the E-aurone unit. Atom labels in this figure follow the numbering adopted for the GIAO calculations and do not correspond to the proton numbering used in Figure 4.

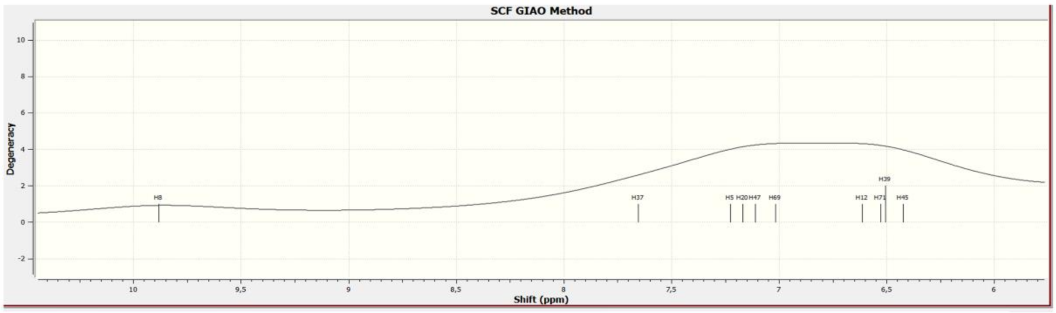

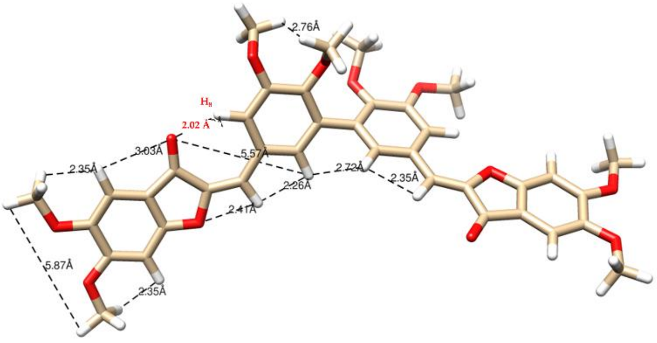

(*E*,*Z*)-**1a**

Figure S14

GIAO-calculated ^{1}H NMR chemical shifts and DFT-optimized geometry of the more extended conformer D of (E,Z)-1a. The diagnostic biphenyl aromatic proton H_8 (δ 10.15 ppm) is located 2.02 Å from the carbonyl oxygen of the E-aurone unit. Atom labels in this figure follow the numbering adopted for the GIAO calculations and do not correspond to the proton numbering used in Figure 4.

Isomer / difference	ΔE SVP	ΔG SVP	ΔG TZVP//SVP	ΔG qRRHO TZVP//SVP
(Z,Z′)-1a: C − A	+6.33	+2.45	+0.90	+2.49
(E,Z)-1a: D − B	+5.40	+1.61	−1.42	+0.15

Table S1.

Relative conformational energies of the investigated bis-aurones.

Open minus compact (C − A for (*Z*,*Z*′)-**1a**; D − B for (*E*,*Z*)-**1a**), in kcal mol^{-1}. Positive values favour the compact conformer. Geometries and vibrational corrections: B3LYP-D3(BJ)/def2-SVP/SMD(chloroform), 298.15 K and 1 atm. The def2-TZVP columns include single-point energy corrections; qRRHO uses a 100 cm^{-1} cutoff. The conformer ordering depends on the basis-set and entropy treatment; consequently, these relative energies alone do not establish a unique preferred solution conformation. Values are reported for the four optimized conformers discussed in the manuscript.
